# NF-κB/NFAT signaling contributes to B7-H3 TRuC-T cell cytotoxicity and supports a reporter platform for glioblastoma immunotherapy

**DOI:** 10.3389/fimmu.2026.1876463

**Published:** 2026-07-30

**Authors:** Junyue Lin, Peiyuan Zhang, Xinyu Li, Shanhong Lu, Qianjin Yu, Jiahao Zheng, Jing Yang, Xuanhao Chen, Cheng Wei, Jimin Gao, Ai Zhao

**Affiliations:** 1 Key Laboratory of Laboratory Medicine, Ministry of Education, School of Laboratory Medicine and Life Sciences, Wenzhou Medical University, Wenzhou, China; 2 Zhejiang Provincial Key Laboratory of Aging and Neurological Disorder Research, The First Affiliated Hospital of Wenzhou Medical University, Wenzhou, China; 3 Zhejiang Qixin Biotech, Wenzhou, China; 4 Department of Geriatric, Affiliated Hangzhou First People’s Hospital, Westlake University School of Medicine, Hangzhou, China

**Keywords:** glioblastoma, immunocyte therapy, NFAT, NF-κB, TRuC-T cells

## Abstract

**Introduction:**

Glioblastoma (GBM), the most prevalent and aggressive malignant tumor of the nervous system, presents a formidable clinical challenge due to its rapid progression and extremely poor prognosis. T cell receptor fusion constructs (TRuC) -T cells represent a highly promising engineered T-cell therapy beyond CAR-T technology. Early prediction of TRuC-T cell cytotoxicity against GBM is crucial for clinical application.

**Methods:**

We developed B7-H3-targeting TRuC-T cells and incorporated NF-κB- or NFAT-responsive transcriptional elements into the TRuC construct. Using flow cytometry, luciferase assays, and *in vitro*/*vivo* functional studies, we evaluated whether TRuC-T cells exert cytotoxicity via NF-κB and NFAT signaling pathways.

**Results:**

B7-H3 TRuC-T cells effectively suppressed GBM growth both *in vitro* and *in vivo*. Co-culture of TRuC-T cells carrying NF-κB- or NFAT-reporters with target cells induced GFP expression, indicating pathway activation.

**Discussion:**

Upon engagement with B7-H3, both NFAT and NF-κB signaling pathways are activated and participate in the regulation of B7-H3-targeting TRuC-T cells. Our data demonstrate that NF-κB/NFAT transcriptional reporters integrated into these TRuC constructs can be utilized to monitor target recognition and may serve as early indicators of cytotoxic efficacy. Overall, this system suggests a potential avenue for GBM therapy and offers an initial framework for the functional assessment of TRuC-T cell therapeutic potency.

## 1 Introduction

Engineered T cells, particularly CAR-T cells, have demonstrated remarkable efficacy in treating hematological malignancies (1, 2). Nevertheless, significant variability in clinical outcomes persists, potentially attributable to factors such as inter-patient differences in target antigen expression levels and inconsistent persistence of the engineered T cells (3, 4). The functional state of engineered T cells, particularly their activation, proliferation, and persistence, is a critical factor influencing clinical efficacy (5, 6). T cell receptor fusion constructs (TRuC) -T cells, which utilize an scFv fused to the CD3ϵ subunit for MHC-independent target recognition, represent a promising engineering platform. While fusing the scFv to multiple TCR-CD3 subunits (TCRα, TCRβ, CD3γ, CD3δ, or CD3ϵ) is possible, functional outcomes are subunit-dependent; CD3ϵ- and CD3γ-based constructs elicit potent and specific activation, with the CD3ϵ configuration being particularly notable for its robust antitumor activity (7). Compared to CAR-T cells, TRuC utilizes the full TCR signaling pathway to transduce signals, offering advantages in tumor killing—such as reduced cytokine secretion and superior cytotoxic activity (8, 9). In a Phase I study of gavo-cel, a mesothelin-targeted TRuC-T cell therapy, objective responses were achieved in patients with refractory solid tumors, alongside a manageable safety profile (10). However, research on the cytotoxic function and regulatory mechanisms of TRuC-T cells remains relatively limited. The NF-κB and NFAT signaling pathways serve as central hubs for signal transduction during T cell activation and are essential for cytokine secretion (e.g., TNF-α, IL-2) and cell survival (11–13). Accordingly, the activation status of these two pathways is considered a key determinant of the therapeutic efficacy of TRuC-T cell therapy. Current methodologies for monitoring the activation of NF-κB and NFAT signaling pathways predominantly rely on Western blot and flow cytometry, which are inherently limited in their capacity to capture real-time signaling dynamics (14). More critically, CAR-T cells often undergo antigen-induced downregulation of surface CAR expression (15–17), complicating the direct assessment of pathway activity using these conventional approaches. Consequently, this technical constraint undermines the feasibility of utilizing signaling pathway activity as a predictive indicator of clinical efficacy.

Glioblastoma (GBM) stands as the most prevalent and aggressive primary malignant tumor of the central nervous system in adults (18, 19). Although CAR-T cell therapy offers a novel therapeutic avenue, its safety remains a critical concern (20, 21). B7-H3 is abundantly expressed on the surface of various tumor cells yet is minimally detected in normal tissues, making it an ideal candidate for targeted cancer therapy (22, 23).

Herein, we constructed B7-H3-targeted TRuC-T cells and systematically evaluated their cytotoxic efficacy against glioblastoma in both *in vitro* and *in vivo* models. Concurrently, we engineered T cells incorporating NF-κB/NFAT reporter systems, and through real-time dynamic monitoring, we confirmed that the anti-tumor activity of B7-H3 TRuC-T cells is contingent upon the activation of these signaling pathways. Based on these findings, we propose a novel assessment strategy that leverages NF-κB/NFAT pathway activation levels to predict the cytotoxic capacity of TRuC-T cells, thereby offering a potential therapeutic paradigm for glioblastoma cell therapy.

## 2 Methods

### 2.1 Plasmid construction and lentivirus production

A lentiviral vector encoding GFP and mCherry was constructed using the pLenti-EF1α-MCS eukaryotic expression vector. The coding sequences for GFP were cloned into the pLenti-EF1α-MCS vector. cDNA fragments encoding NF-κB-G(F), NF-κB-G(R), NFAT-G(F), and NFAT-G(R)—each containing NF-κB transcription factor, NFAT transcription factor, a mini promoter, and GFP—were synthesized by Genewiz (Suzhou, China). These fragments were then cloned upstream of the EF1α promoter in a lentiviral vector expressing mCherry, yielding the final constructs: NF-κB-G-M(F), NF-κB-G-M(R), NFAT-G-M(F), and NFAT-G-M(R). In parallel, the NF-κB-G(F) and NFAT-G(F) fragments were cloned upstream of the EF1α promoter within the B7-H3 TRuC lentiviral backbone, yielding NF-κB-G-B7 and NFAT-G-B7, respectively. The B7-H3 TRuC construct was composed of a CD8α signal peptide, an anti-B7-H3 single-chain variable fragment (scFv) (24), a (G_4_S)_4_ linker, and the full-length CD3ϵ chain (including extracellular, transmembrane, and cytoplasmic domains) arranged in tandem. Subsequent lentiviral production was performed in HEK-293T cells (ATCC, Manassas, VA, USA) employing a third-generation packaging system, which included pLP1, pLP2, and pMD2.G. The nucleotide sequences encoding the NF-κB transcription factor, NFAT transcription factor, mini-promoter, and B7-H3 TRuC are provided in the **Supplementary Data 1**.

### 2.2 Cell lines and cell culture

Human cell lines, including HEK-293T, U-87, U-251, LN-229, A172 and KNS89, were sourced from the American Type Culture Collection (ATCC; Manassas, VA, USA). To enable bioluminescence imaging, U-87 and U-251 cells were transduced with a luciferase-encoding lentivirus to establish U-87-Luc and U-251-Luc stable lines. All cells were maintained in DMEM containing 10% fetal bovine serum (FBS) (PAN, ST30-3302) under standard culture conditions (37 °C, 5% CO_2_).

### 2.3 Generation of TRuC-T cells

Peripheral blood mononuclear cells (PBMCs) were isolated from the whole blood of healthy donors by Ficoll density gradient centrifugation. T cells were subsequently stimulated with anti-human CD3/CD28 magnetic beads (Thermo,11141D) at a 1:1 bead-to-cell ratio and cultured at a density of 1 × 10^6^ cells/mL in KBM581 serum-free medium (Cas#88-581-CM, Corning) supplemented with IL-2 (100 IU/mL) (Cas#200-02-10UG, Peprotech). Following 24–48 hours of activation, T cells were transduced with lentiviral particles. Transduction efficiency was assessed on day 7 post-transduction.

### 2.4 Flow cytometry and antibodies

B7-H3 expression levels on tumor cells were determined by flow cytometry using APC-conjugated anti-human B7-H3 antibody (Clone MIH42, Cas#351005, BioLegend). For detection of surface anti-B7-H3 scFv on TRuC-T cells, cells were stained with biotinylated recombinant human B7-H3 protein (Cas#B73-H82E6, ACRO Biosystems), subsequently labeled with APC-streptavidin (Cas#16902, BioLegend).

Immunophenotyping of TRuC-T cells was performed with antibodies against CD4 (FITC, Clone OKT4, Cas#317407, BioLegend), CD8a (PerCP, Clone HIT8a, Cas#300922; or PE, Clone RPA-T8, Cas#301064, BioLegend), CD62L (PE, Clone DREG-56, Cas#304805, BioLegend), and CD45RO (APC,Clone UCHL1, Cas#304210, BioLegend), all from BioLegend. Activation status was monitored via upregulation of CD25 (FITC/APC,Clone M-A251, Cas#356105/356109, BioLegend) and CD69 (PerCP/APC, Clone FN50, Cas#310927/310909, BioLegend).

In the intracellular cytokine staining assay, TRuC-T cells were co-incubated with target cells at an effector-to-target (E:T) ratio of 5:1 for 1 hour. Brefeldin A (BFA) (Cas#HY-16592,MCE) was then added to block cytokine secretion from TRuC-T cells, and the co-incubation was continued for an additional 5 hours. Following incubation, the cells were fixed with 4% paraformaldehyde and subsequently permeabilized using a permeabilization buffer (Cas#AKES085,BioLegend). The permeabilized TRuC-T cells were then stained with fluorochrome-conjugated antibodies against tumor necrosis factor-alpha (TNF-α) (APC, Clone MAb11,Cas#502912 BioLegend), interferon-gamma (IFN-γ) (PE, Clone B27, Cas#506507, BioLegend), and granzyme B (GZMB) (PE,Clone QA18A28, Cas#396405, BioLegend) for 30 minutes at the appropriate temperature. After washing, the expression levels of these intracellular effectors were evaluated by flow cytometry.

Flow cytometric analysis was conducted on a FACS Aria II instrument (BD Biosciences), and data were processed with FlowJo v10 software (FlowJo, Ashland, OR, USA).

### 2.5 Cytokine analysis

The levels of secreted cytokines and chemokines were determined by enzyme-linked immunosorbent assay (ELISA). Following a 24-hour co-culture of B7-H3 TRuC-T cells (0.5 million) with U-87 or U-251 target cells (0.1 million) at an effector-to-target (E:T) ratio of 5:1, culture supernatants were harvested and subjected to ELISA for quantification of IL-2 (Cas#EK102, MULTI SCIENCES), IFN-γ (Cas#EK180, MULTI SCIENCES), IL-6 (Cas#EK106, MULTI SCIENCES), GZMB (Cas#EK158, MULTI SCIENCES) and TNF-α (Cas#EK182, MULTI SCIENCES).

### 2.6 Cytolytic toxicity assay

Luciferase-expressing target cells were plated in 96-well culture plates 24 hours before the cytotoxicity assay. Subsequently, effector cells were added at designated effector-to-target (E:T) ratios and co-cultured with target cells for 24 hours; Mock-T cells were included as a control. Maximum luminescence (Kmax) was determined from target cells cultured alone (negative control), while minimum luminescence (Kmin) was obtained from target cells completely lysed with ddH_2_O (positive control). After incubation, D-luciferin (Cas#2591-17-5, MedChemExpress) was added to each well at a final concentration of 0.5 mM, and luminescence values (K) were recorded with a microplate reader. The specific lysis rate was calculated as follows: (Kmin - K)/(Kmin - Kmax) × 100%.

### 2.7 TRuC internalization

Live B7-H3 TRuC-GFP T cells were labeled with Hoechst 33342 (10 min, 37 °C), washed twice with PBS, and then incubated with biotinylated B7-H3 protein (30 min, 4 °C). After two washes, the cells were stained with Alexa Fluor 647-streptavidin (Cas#7516-1mL, BioLegend) secondary antibody (30 min, 4 °C), washed twice again, and resuspended in culture medium. The cell suspension was split into two fractions: one was incubated at 37 °C for 1 h to allow internalization, and the other was kept at 4 °C as a control. Cells were then imaged by confocal microscopy to detect intracellular red fluorescence, indicating B7-H3 protein localization.

### 2.8 Animal studies

To assess antitumor efficacy *in vivo*, U-87-luc cells were subcutaneously inoculated into 4- to 6-week-old NOG mice. Tumor engraftment was confirmed seven days later using an *In Vivo* Imaging System (IVIS). Tumor-bearing mice were then randomly assigned to two groups and received either Mock-T cells (negative control) or B7-H3 TRuC-T cells via tail vein injection. Tumor growth was monitored periodically by IVIS imaging throughout the study. The care and handling of the mice, along with the experimental procedures, were carried out in strict compliance with the guidelines sanctioned by the Animal Ethics Committee of Wenzhou Medical University.

### 2.9 Real-time quantitative reverse transcription-PCR

Total RNA was extracted using the RNeasy Kit (Cas#MS102, NCM) and reverse-transcribed into cDNA using PrimeScript RT Master Mix (Cas#RT333-01,Vazyme). Quantitative PCR was performed using SYBR qPCR Master Mix (Cas#Q412-02, Vazyme). The cycling protocol was 95 °C for 30 s, followed by 40 cycles of 95 °C for 5 s and 60 °C for 30 s. Gene expression was calculated using the 2−ΔΔCt method and normalized to GAPDH. All reactions were performed in triplicate.

The following primer pairs were used:

GAPDH forward: 5′-GTCTCCTCTGACTTCAACAGCG-3′
GAPDH reverse: 5′-ACCACCCTGTTGCTGTAGCCAA-3′
WPRE forward: 5′-CCTTTCCGGGACTTTCGCTTT-3′
WPRE reverse: 5′-GCAGAATCCAGGTGGCAACA-3′

### 2.10 Statistical analysis

All data are presented as the mean ± standard deviation (SD). Statistical analyses were performed using GraphPad Prism 9.0 (GraphPad Software, San Diego, CA, USA). Comparisons between two groups were conducted using unpaired two-tailed Student’s t-tests, while one-way or two-way ANOVA was applied for multiple group comparisons, as appropriate. Survival differences were analyzed using the log-rank Mantel–Cox test. Statistical significance was defined as follows: ns, not significant; *p < 0.05; **p < 0.01; ***p < 0.001; ****p < 0.0001. All experiments were independently repeated at least three times.

## 3 Results

### 3.1 Generation of B7-H3 TRuC-T cells

**Figure 1:**
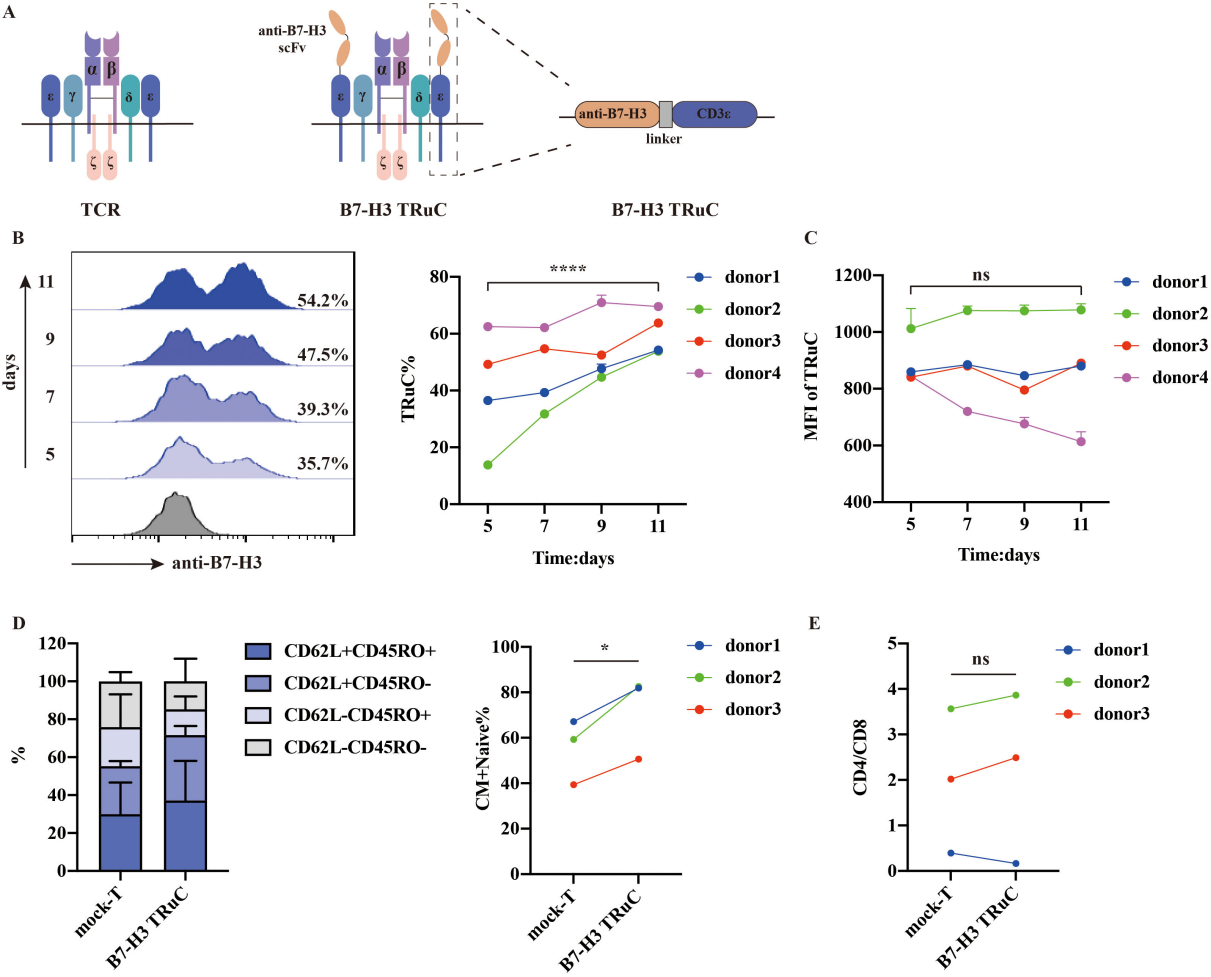
Generation and characterization of B7-H3 TRuC-T cells. **(A)** Schematic diagram of the B7-H3 TRuC construct, in which an anti-B7-H3 single-chain variable fragment (scFv) is directly linked to human CD3ε via a flexible (G_4_S)_4_ linker. **(B)** Flow cytometric assessment of B7-H3 TRuC-T cell generation efficiency on days 5, 7, 9, and 11 post-transduction (n = 4 independent donors). **(C)** Statistical analysis of the mean fluorescence intensity (MFI) of TRuC surface expression across different donors over time (n = 4 independent donors; each dot represents an independent donor). **(D)** Representative flow cytometry plots showing T cell differentiation subsets. Central memory T cells (Tcm) were defined as CD45RO^+^CD62L^+^, while naïve T cells were defined as CD45RO^-^CD62L^+^ (n = 3 independent donors). **(E)** Quantification of CD4^+^ and CD8^+^ T cell subsets in B7-H3 TRuC-T cells by flow cytometry (n = 3 independent donors; each dot represents an independent donor). Results are expressed as mean ± SD. Using two-way ANOVA with Tukey’s multiple comparisons test, we examined the statistical significance of A and D, and one-way ANOVA with Tukey’s multiple comparisons test for B and **(C)** Paired T test to test for significance in C and **(D)** Statistical significance levels are indicated as follows: *p <0.05, **p < 0.01, ***p < 0.001, ****p < 0.0001.

To construct the B7-H3 TRuC lentiviral vector, a single-chain variable fragment (scFv) targeting human B7-H3 was fused to human CD3ε via a (G4S)_4_ linker (**Figure 1A**). Following lentiviral production and transduction of activated T cells, the generation efficiency of B7-H3 TRuC-T cells was assessed by flow cytometry on days 5, 7, 9, and 11 post-transduction (**Figure 1B**). The results demonstrated a progressive increase in the percentage of B7-H3 TRuC-expressing T cells over time, whereas the mean fluorescence intensity (MFI) of surface TRuC expression remained relatively stable without significant differences(**Figure 1C**). Given that the differentiation status of CAR-T cells is a key determinant of therapeutic efficacy, we further characterized the differentiation subsets of B7-H3 TRuC-T cells. Notably, B7-H3 TRuC-T cells exhibited a higher proportion of naïve and central memory T (Tcm) cells compared to Mock-T cells (**Figure 1D**), while the CD4/CD8 ratio showed no significant alteration (**Figure 1E**).

### 3.2 B7-H3 TRuC-T cells effectively inhibit glioblastoma cells *in vitro* and *in vivo*

**Figure 2:**
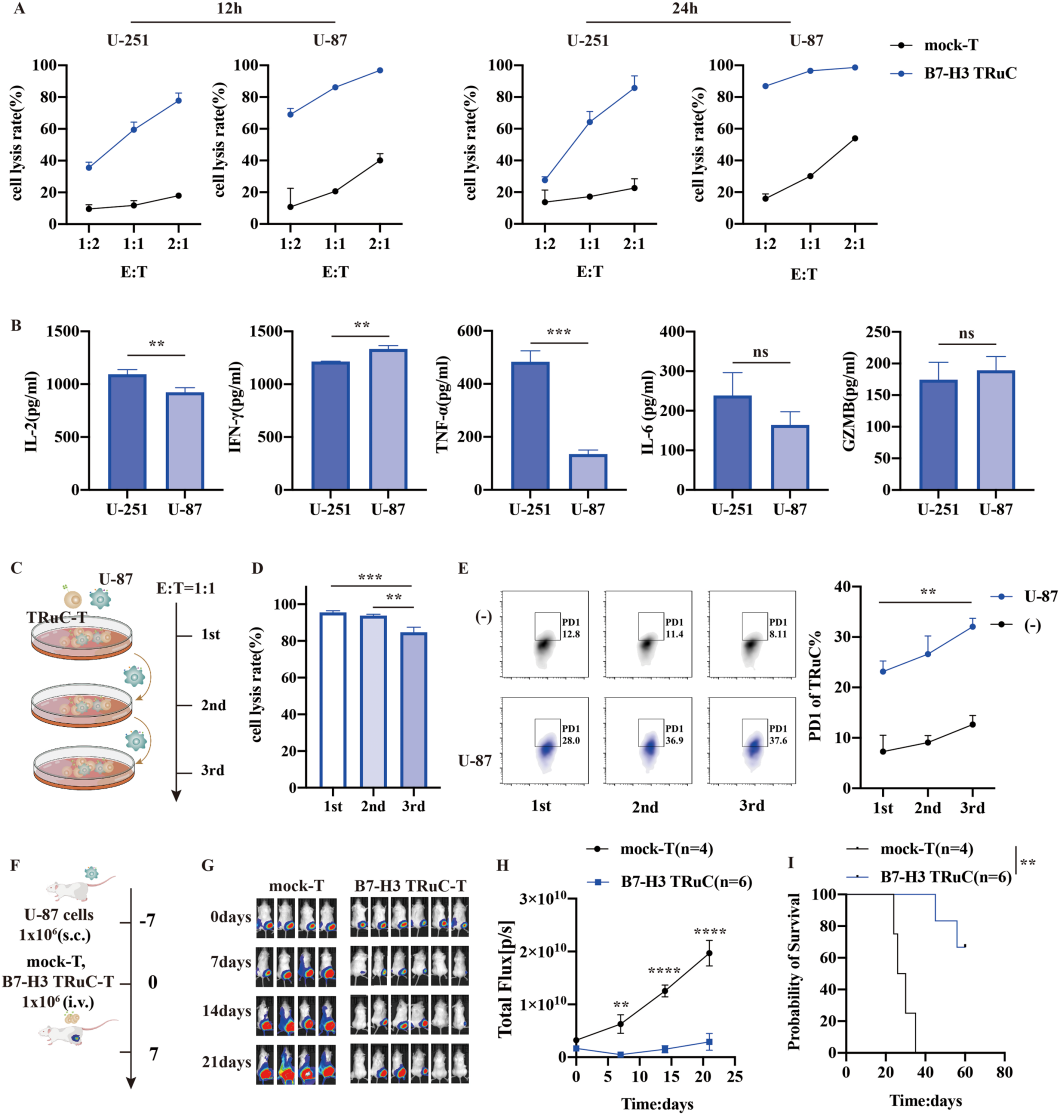
B7-H3 TRuC-T cells exhibit potent cytotoxicity against glioblastoma cells *in vitro* and *in vivo*. **(A)** Cytotoxic activity of B7-H3 TRuC-T cells against U-87-Luc and U-251-Luc target cells assessed by luciferase-based bioluminescence assay following 12 or 24 hours of co-culture at indicated effector-to-target (E:T) ratios(n=3). **(B)** Cytokine secretion profiles of IL-2, IFN-γ, IL-6, GZMB and TNF-α from B7-H3 TRuC-T cells co-cultured with U-251 or U-87 cells, measured by ELISA(n=3). **(C)** Schematic representation of the sequential stimulation assay. B7-H3 TRuC-T cells were initially co-incubated with U-87 cells at an effector-to-target ratio of 1:1, followed by repeated stimulation with fresh U-87 cells on days 2, 4, and 6. At each time point, the lytic capacity against U-87 target cells and the expression of surface PD-1 on TRuC-T cells were measured. **(D)** Quantitative assessment of the cytolytic activity of B7-H3 TRuC-T cells against U-87 cells at the indicated time points(n=3). **(E)** Surface PD-1 expression levels on B7-H3 TRuC-T cells after co-incubation with U-87 cells (n=3). **(F)** Schematic illustration of the *in vivo* experimental design. NOG mice (6–8 weeks old) were subcutaneously inoculated with 1 × 10^6^ U-87-Luc cells, followed by intravenous injection of 1 × 10^6^ TRuC-T cells via the tail vein one week later. Tumor growth was monitored weekly using an IVIS imaging system. **(G)** Representative IVIS images showing U-87-Luc tumor growth in mice over time(mock-T n=4, B7-H3 TRuC n=6). **(H)** Quantification of bioluminescence signal intensity(mock-T n=4, B7-H3 TRuC n=6). **(I)** Kaplan-Meier survival curves for mice in each treatment group(mock-T n=4, B7-H3 TRuC n=6). All experiments were performed in three independent biological replicates. Results are expressed as mean ± SD. Using two-way ANOVA with Tukey’s multiple comparisons test, we examined the statistical significance of A, E and H, unpaired t test to test for significance in B, one-way ANOVA with Tukey’s multiple comparisons test for D and the log-rank (Mantel-Cox) test to assess significance in **(I)** *p <0.05, **p < 0.01, ***p < 0.001, ****p < 0.0001.

To assess whether B7-H3 TRuC-T cells could specifically lyse B7-H3-positive targets, GSCs were co-cultured with either Mock-T or B7-H3 TRuC-T cells for 48 hours. Microscopic examination revealed pronounced lysis of GSCs in the presence of B7-H3 TRuC-T cells, whereas GSCs co-cultured with Mock-T cells retained intact morphology. Consistent with this, flow cytometric analysis demonstrated a significantly higher proportion of viable T cells in the B7-H3 TRuC-T group following co-culture (**Supplementary Figure 1A**). We further generated U-87 and U-251 cell lines stably expressing firefly luciferase (U-87-Luc and U-251-Luc) to quantitatively assess cytotoxicity. B7-H3 TRuC-T cells were co-cultured with U-87-Luc or U-251-Luc target cells at effector-to-target (E:T) ratios of 1:2, 1:1, and 2:1 for 12 or 24 hours, and target cell lysis was measured using a luciferase-based bioluminescence assay. B7-H3 TRuC-T cells exhibited potent cytotoxicity against both U-87-Luc and U-251-Luc cells, with U-87-Luc cells demonstrating greater susceptibility to elimination (**Figure 2A**). Furthermore, we examined the secretion profiles of IL-2, IL-6, granzyme B (GZMB), IFN-γ, and TNF-α following co-culture with U-87 or U-251 cells. Co-incubation with U-251 cells significantly augmented the release of IL-2 and TNF-α; however, the levels of IL-6 and GZMB in the co-culture supernatants remained largely unchanged (**Figure 2B**). Given that persistent antigenic stimulation typically drives T-cell functional exhaustion, we exposed B7-H3 TRuC-T cells to three consecutive rounds of U-87 stimulation (**Figure 2C**). Upon evaluating their cytolytic activity against U-87 cells, we observed a pronounced reduction in killing efficiency after the third co-incubation (**Figure 2D**). Consistent with this functional decline, surface PD-1 expression was significantly elevated following the third round of stimulation (**Figure 2E**). To evaluate the antitumor efficacy of B7-H3 TRuC-T cells *in vivo*, NOG mice were subcutaneously inoculated with 1 × 10^6^U-87-Luc cells and subsequently treated with B7-H3 TRuC-T cells seven days after tumor implantation (**Figure 2F**). Mice receiving a single dose of 1 × 10^6^ B7-H3 TRuC-T cells exhibited significantly reduced tumor burden compared to control mice (**Figures 2G, H**), accompanied by a marked survival advantage (**Figure 2I**).

### 3.3 Construction of B7-H3-targeted TRuC-T cells incorporating NF-κB or NFAT response elements

**Figure 3:**
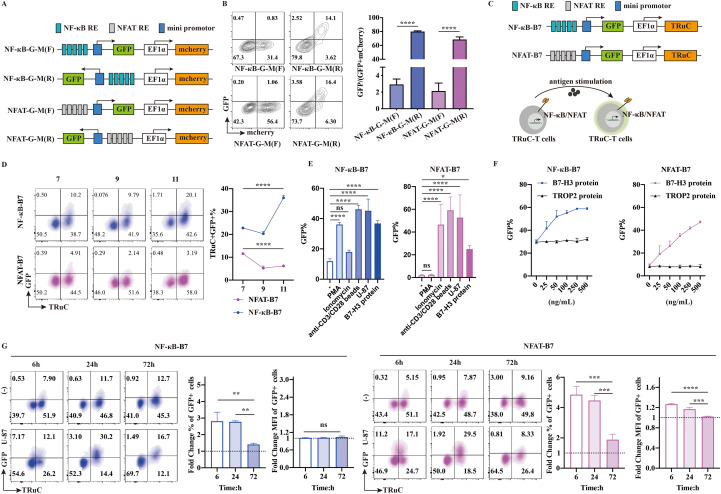
Successful generation of B7-H3 TRuC-T cells incorporating NF-κB or NFAT response elements. **(A)** Schematic representation of the constructs containing NF-κB or NFAT response elements. GFP expression is driven by the NF-κB or NFAT response elements, whereas mCherry is constitutively expressed under the control of the EF-1α promoter. Two configurations were designed: one with both promoters in the same orientation and the other with opposing orientations. **(B)** T cells were transduced with the packaged lentiviruses, and on day 9, the expression of GFP and mCherry was assessed by flow cytometry(Data in B are from three technical replicates and are representative of at least three independent donors). **(C)** Schematic diagram of the B7-H3 TRuC vectors incorporating NF-κB or NFAT response elements. **(D)** Transduction efficiency of B7-H3 TRuC-T cells was evaluated by flow cytometry on day 7, 9 and 11 post-transduction(Data in D are from three technical replicates and are representative of at least three independent donors). **(E)**. NFAT-B7 and NF-κB-B7 TRuC-T cells were co-cultured for 6 hours with PMA(25ng/ml), Inocomycin(1μm), anti-CD3/CD28 beads(1:1), U-87(E:T=3:1),B7-H3 protein(250ng/ml). GFP expression was then quantified by flow cytometry. **(F)** NFAT-B7 and NF-κB-B7 TRuC-T cells were co-cultured for 6 hours with B7-H3 or TROP2 protein at concentrations ranging from 0 to 500 ng/mL (0, 25, 50, 100, 250, and 500 ng/mL). GFP expression was then quantified by flow cytometry. **(G)** Expression of TRuC and GFP in NFAT-B7-TRuC-T and NF-κB-B7-TRuC-T cells at 6, 24, and 72 h after co-incubation with U-87 cells at an effector-to-target ratio of 3:1. Data in **(E–G)** were performed in three independent biological replicates. Data are presented as mean ± SD. One-way ANOVA with Tukey’s multiple comparisons test to test for significance in **(B, E–G)** and Two-way ANOVA with Tukey’s multiple comparisons test was used to test for statistical significance in **(D)** *p < 0.05, **p < 0.01, ***p < 0.001, ****p < 0.0001.

To date, numerous canonical DNA sequences capable of inducing activation in human T cells and B cells have been reported, most of which contain multiple tandem binding sites for NF-κB and NFAT. To construct reporters capable of monitoring the activation of NF-κB and NFAT signaling pathways, we designed dual-promoter expression vectors in which the EF-1α promoter drives constitutive expression of mCherry, while an inducible promoter containing NF-κB or NFAT response elements controls GFP expression upon pathway activation (**Figure 3A**). T cells were transduced with four different lentiviral constructs—NF-κB-G-M(F), NF-κB-G-M(R), NFAT-G-M(F), and NFAT-G-M(R)—and analyzed by flow cytometry on day 9 for mCherry and GFP expression. Notably, in the absence of exogenous stimulation, T cells transduced with NF-κB-G-M(R) or NFAT-G-M(R), in which the inducible promoter is oriented opposite to the EF-1α promoter, exhibited basal activation of the minimal promoter, leading to GFP expression. In contrast, T cells transduced with NF-κB-G-M(F) or NFAT-G-M(F), where the inducible promoter is in the same orientation as EF-1α, showed negligible GFP expression, with only mCherry detected (**Figure 3B**). Based on these findings, we generated B7-H3-targeted TRuC constructs incorporating NF-κB or NFAT response elements, designated NF-κB-B7 and NFAT-B7 (**Figure 3C**). Following lentiviral transduction, T cells were assessed by flow cytometry on day 7, 9 and 11 for transduction efficiency. Both NF-κB-B7 TRuC-T and NFAT-B7 TRuC-T cells displayed successful transgene expression; however, GFP expression in NFAT-B7 TRuC-T cells declined over time in culture (**Figure 3D**). Upon stimulation with PMA, Ionomycin, anti-CD3/CD28 beads, U-87 cells, or the B7-H3 protein, NF-κB-B7 and NFAT-B7 TRuC-T cells exhibited distinct responsiveness: GFP expression was robustly induced in NF-κB-B7 TRuC-T cells by PMA, anti-CD3/CD28 beads, U-87 cells, and the B7-H3 protein, whereas in NFAT-B7 TRuC-T cells, it was effectively induced by Ionomycin, anti-CD3/CD28 beads, U-87 cells, and the B7-H3 protein (**Figure 3E**). In contrast, when co-incubated with astrocytes and endothelial cells, the B7-H3 TRuC-T cells carrying NF-κB/NFAT transcriptional elements did not induce detectable GFP expression (**Supplementary Figure 2**). To verify that NF-κB-B7 and NFAT-B7 TRuC-T cells are specifically activated by the B7-H3 antigen, we employed a co-culture system using either B7-H3 or TROP2 protein. Our results demonstrate that GFP expression in these TRuC-T cells is induced specifically by B7-H3, but not by the control antigen TROP2 (**Figure 3F**). We further characterized the temporal dynamics of GFP expression in NF-κB-B7 TRuC-T cells and NFAT-B7 TRuC-T cells upon a single co-incubation with U-87 cells. The induced GFP signal returned to background levels by 72 h post-stimulation (**Figure 3G**).

To investigate whether the introduction of NF-κB and NFAT transcriptional elements and the induced expression of GFP affect the functional properties of B7-H3 TRuC-T cells, We first compared the transduction efficiency of the two constructs in T cells and observed no significant difference (**Supplementary Figure 3A**). Likewise, the expression levels of WPRE mRNA derived from the lentiviral vector showed no notable disparity between the three groups (**Supplementary Figure 3B**), and we evaluated the cytotoxic activity by co-incubating TRuC-T cells with target U-87 cells, and observed no notable difference in killing efficiency (**Supplementary Figure 3C**). Subsequently, we examined their proliferative capacity. The results showed that this modification did not significantly impact cell proliferation (**Supplementary Figure 3D**). To further evaluate functional persistence under conditions of repeated antigen exposure, we co-incubated NFAT-B7 TRuC-T cells and NF-κB-B7 TRuC-T cells with U-87 target cells at a 1:1 effector-to-target ratio in sequential rounds of stimulation (**Supplementary Figure 3E**). After the third round of target addition, we observed a marked reduction in killing efficiency (**Supplementary Figure 3F**), a gradual decline in proliferation rate (**Supplementary Figure 3G**), and a substantial upregulation of surface PD-1 expression (**Supplementary Figure 3H**). However, these changes did not differ significantly between the NFAT/NF-κB-expressing TRuC-T cells and the conventional B7-H3 TRuC-T cells. In parallel, the inducibility of GFP expression in both NFAT-B7 and NF-κB-B7 TRuC-T cells also decreased upon repeated stimulation (**Supplementary Figure 3I**). Collectively, these findings indicate that the introduction of NFAT or NF-κB transcriptional elements does not compromise the overall functionality of B7-H3 TRuC-T cells.

### 3.4 B7-H3 TRuC-T cells mediate tumor cell killing through NF-κB and NFAT signaling pathways

**Figure 4:**
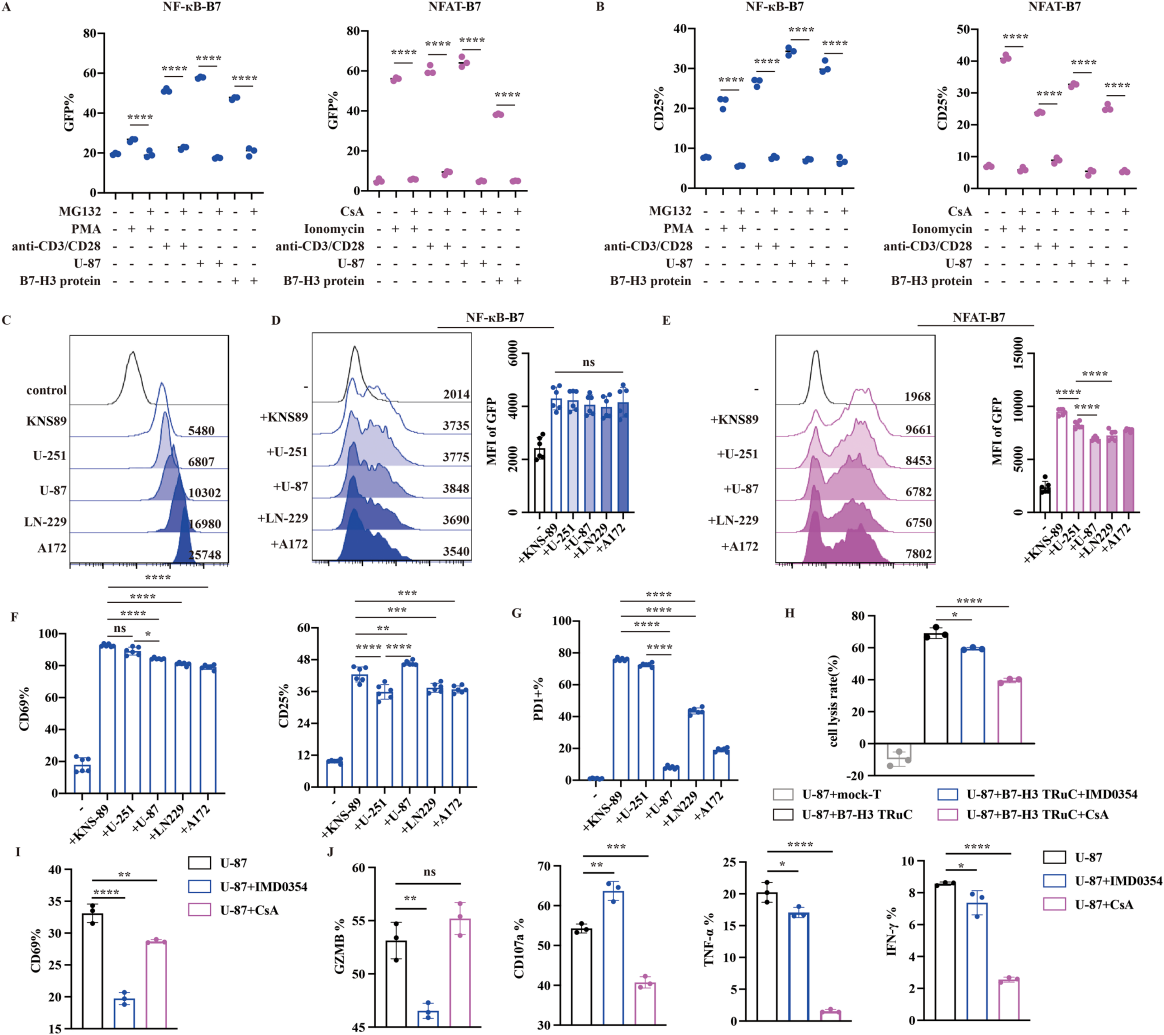
GBM cells with varying B7-H3 expression levels differentially modulate NFAT pathway activation in B7-H3 TRuC-T cells. **(A, B)** NF-κB-B7-T reporter cells were co-incubated with PMA, anti-CD3/CD28, U-87 cells, or B7-H3 protein for 6 hours, with or without MG132 treatment. NFAT-B7-T reporter cells were co-incubated with Ionomycin, anti-CD3/CD28, U-87 cells, or B7-H3 protein for 6 hours, with or without CsA treatment. **(A)** GFP expression was assessed by flow cytometry. **(B)** CD25 expression was evaluated by flow cytometry under the indicated conditions (Data in A and B are from three technical replicates and are performed in three independent donors). **(C)** Surface B7-H3 expression on KNS89, U-251, U-87, LN229, and A172 cells was detected by flow cytometry. Data are presented as mean fluorescence intensity (MFI) of B7-H3. **(D)** NF-κB-B7-T reporter cells were co-incubated with KNS89, U-251, U-87, LN-229, or A172 cells for 6 hours. GFP expression is shown as representative flow cytometry plots (left) and quantification of mean fluorescence intensity (right) (n = 3,The experiment were repeated independently at least three times). **(E)** NFAT-B7-T reporter cells were co-incubated with the indicated GBM cell lines for 6 hours. GFP expression was analyzed by flow cytometry, with representative histograms shown on the left and quantification of MFI on the right (n = 3,The experiment were repeated independently at least three times). **(F)** B7-H3 TRuC-T cells were co-incubated with KNS89, U-251, U-87, LN-229, or A172 cells for 6 hours. Surface expression of CD25 and CD69 on TRuC-T cells was measured by flow cytometry (n = 3,The experiment were repeated independently at least three times). **(G)** B7-H3 TRuC-T cells were co-incubated with the indicated GBM cell lines for 72 hours. PD-1 expression on the surface of TRuC-T cells was assessed by flow cytometry(n = 3,The experiment were repeated independently at least three times). **(H)** TRuC-T cells were co-incubated with U-87 at an E:T ratio of 1:2 for 24 h, and the inhibitory effects of IMD0354 and CsA on cytotoxicity were measured by luciferase assay (n = 3,n represents the number of independent experiments). **(I)** After co-incubation at 5:1 for 6 h, surface CD69 expression on TRuC-T cells was analyzed by flow cytometry (n = 3, independent experiments). **(J)** Following co-incubation at 5:1 for 6 h, expression of CD107a, TNF-α, IFN-γ, and GZMB in TRuC-T cells was determined by flow cytometry (n = 3,independent experiments).Data are presented as mean ± SD. One-way ANOVA with Tukey’s multiple comparisons test was used to test for statistical significance in **(A, B, D, E–J)** *p < 0.05, **p < 0.01, ***p < 0.001, ****p < 0.0001.

We utilized PMA and Ionomycin to stimulate NF-κB-B7-T and NFAT-B7-T reporter cells, respectively. PMA specifically activated NF-κB-B7-T cells, inducing GFP expression, which was abrogated by MG132. In contrast, Ionomycin selectively activated NFAT-B7-T cells, leading to GFP expression, an effect inhibited by CsA (**Figure 4A**). Furthermore, co-incubation with U-87 cells or B7-H3 protein resulted in the activation of both NF-κB-B7-T and NFAT-B7-T cells, as evidenced by upregulated CD25 expression, which was effectively suppressed by MG132 or CsA, respectively. These results demonstrate that the activation of B7-H3 TRuC-T cells is coordinately regulated by both NF-κB and NFAT signaling pathways (**Figure 4B**). Given the heterogeneous expression of surface antigens among tumor cell lines, we profiled B7-H3 expression across five distinct GBM cell lines. Flow cytometric analysis revealed that KNS89 cells exhibited the lowest surface B7-H3 expression, whereas A172 cells displayed the highest level (**Figure 4C**). To delineate the differential engagement of NF-κB and NFAT signaling pathways in B7-H3 TRuC-T cells upon encountering various GBM targets, we co-cultured B7-H3 TRuC-T cells with the indicated GBM cell lines for 6 hours and subsequently assessed GFP expression in the reporter systems. Co-incubation with different GBM cells induced comparable levels of GFP expression in NF-κB-B7-T reporter cells (**Figure 4D**). Notably, KNS89 cells elicited the highest GFP expression in NFAT-B7-T reporter cells (**Figure 4E**). Consistent with these observations, analysis of activation marker expression on B7-H3 TRuC-T cells after 6 hours of co-incubation revealed distinct profiles: CD69 expression was most pronounced on TRuC-T cells co-cultured with KNS89 cells, while CD25 expression peaked in the U-87 cell co-culture group (**Figure 4F**). Given that sustained NFAT signaling has been implicated in CAR-T cell exhaustion, we examined PD-1 expression on B7-H3 TRuC-T cells following 72 hours of co-incubation with the GBM cell lines. PD-1 expression was highest on TRuC-T cells exposed to KNS89 cells and lowest on those exposed to U-87 cells (**Figure 4G**), mirroring the pattern of NFAT pathway activation observed in the reporter assays.

To further investigate whether the NF-κB and NFAT pathways partially mediate the cytotoxic function of B7-H3 TRuC-T cells, TRuC-T cells were pretreated with IMD0354 (an NF-κB inhibitor) and CsA, respectively, and subsequently co-incubated with U-87 cells. The results showed that both inhibitors partially attenuated the cytotoxic activity of TRuC-T cells against U-87(**Figure 4H**), accompanied by a significant reduction in CD69 expression following co-incubation, indicating that T-cell activation was impaired(**Figure 4I**). Mechanistically, IMD0354 significantly downregulated the expression of GZMB, whereas CsA predominantly reduced the level of the degranulation marker CD107a. In addition, both inhibitors markedly suppressed the secretion of IFN-γ and TNF-α(**Figure 4J**). Collectively, these findings suggest that the NF-κB and NFAT pathways play regulatory roles in the cytotoxic function of TRuC-T cells. More importantly, the vector platform harboring NF-κB or NFAT transcriptional elements may serve as an effective functional predictor for evaluating the target recognition and killing efficacy of B7-H3 TRuC-T cells.

### 3.5 Upon co-culture with target cells, B7-H3 TRuC-T cells undergo degradation via both proteasomal and lysosomal pathways

**Figure 5:**
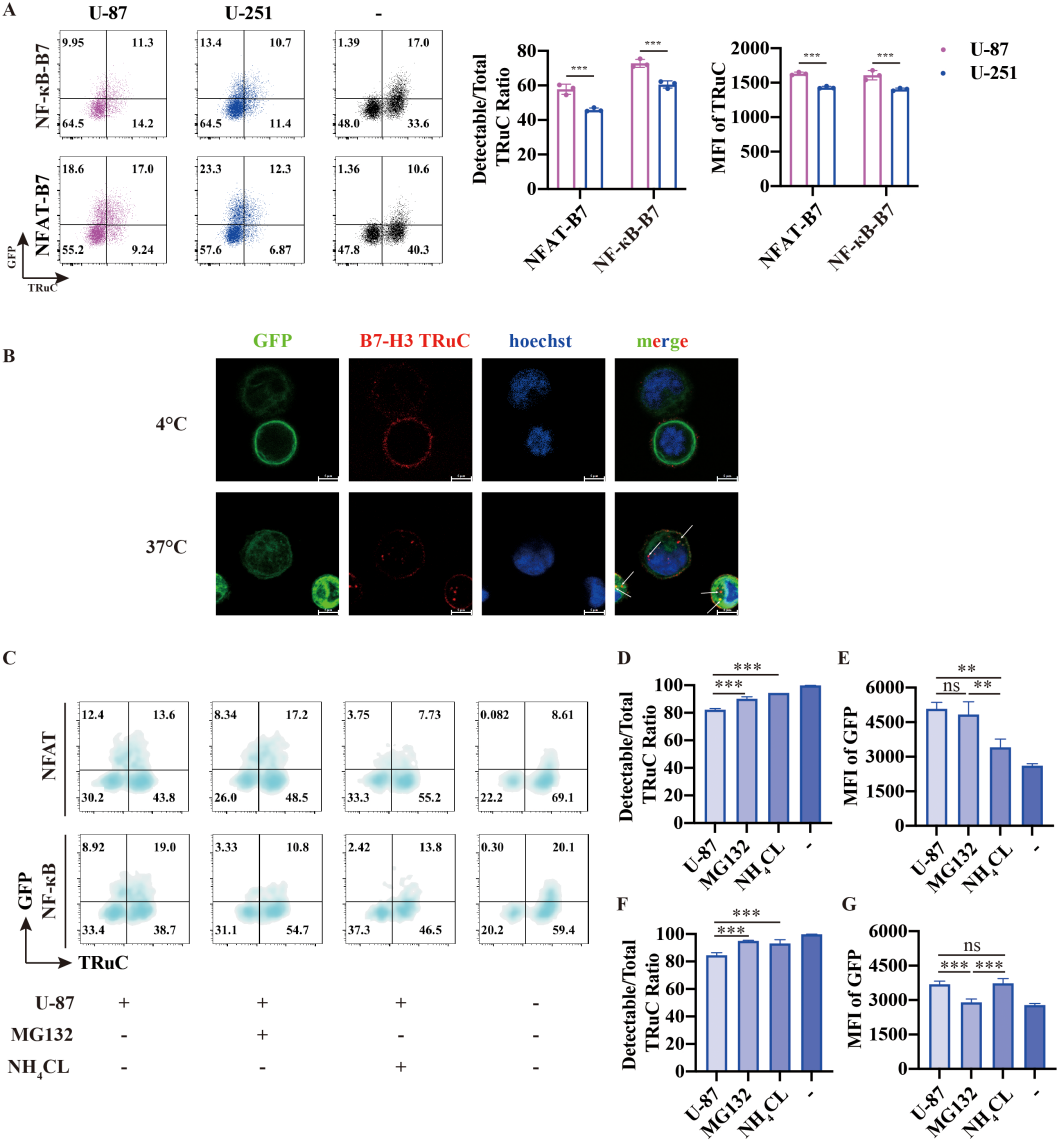
B7-H3 TRuC-T cells downregulate TRuC expression through internalization and degradation, as visualized by NF-κB/NFAT-driven GFP reporters. **(A)** B7-H3 TRuC-T cells were co-cultured with U-87 or U-251 for 6 hours, TRuC expression efficiency and GFP were assessed by flow cytometry(n=3,independent experiments). **(B)** A B7-H3 TRuC-GFP fusion construct was generated, and internalization of B7-H3 TRuC was evaluated by confocal microscopy. **(C)** B7-H3 TRuC-T cells were co-cultured with target cells for 6 hours in the presence or absence of NH_4_Cl or MG132, and the percentage of TRuC-positive cells was determined by flow cytometry. **(D)** Quantification of detectable TRuC+ cells among NFAT-B7 TRuC-T cells under the indicated conditions, relative to unstimulated controls (n = 3,independent experiments). **(E)** Quantification of NFAT-driven GFP expression under the indicated conditions(n = 3,independent experiments). **(F)** Quantification of detectable TRuC+ cells among NF-κB-B7 TRuC-T cells under the indicated conditions, relative to unstimulated controls(n = 3). **(G)** Quantification of NF-κB-driven GFP expression under the indicated conditions(n = 3,independent experiments). Data are presented as mean ± SD. Two-way ANOVA with Tukey’s multiple comparisons test was used to test for statistical significance in **(A)**, one-way ANOVA with Tukey’s multiple comparisons test was used to test for statistical significance in **(D–G)** *p < 0.05, **p < 0.01, ***p < 0.001, ****p < 0.0001.

Following antigen stimulation, CAR-T cells exhibit a loss of surface CAR expression. We observed that TRuC expression on the surface of TRuC-T cells was also reduced after 6 hours of co-culture with U-87 or U-251 cells. Interestingly, U-251 cells accelerate the loss of TRuC from the surface of B7-H3 TRuC-T cells (**Figure 5A**). To investigate whether antigen engagement induces internalization of TRuC, we generated B7-H3 TRuC-GFP T cells with direct GFP fusion (**Supplementary Figure 4**). Then, we incubated B7-H3 TRuC-GFP-T cells with recombinant B7-H3 protein. This revealed that B7-H3 protein was internalized into the cells following incubation at 37 °C (**Figure 5B**). To further delineate the degradation pathways involved, we co-cultured NF-κB-B7 TRuC-T and NFAT-B7 TRuC-T cells with U-87 cells in the presence or absence of MG132 (proteasome inhibitor) or NH_4_Cl (lysosome inhibitor). Co-culture with target cells alone resulted in a distinct population of TRuC-GFP+ cells with reduced surface TRuC expression, confirming antigen-induced TRuC loss (**Figure 5C**). Notably, both MG132 and NH_4_Cl partially attenuated the loss of surface TRuC (**Figures 5D, F**). Interestingly, NH_4_Cl treatment significantly inhibited GFP expression in NFAT-B7 TRuC-T cells, whereas MG132 had no apparent effect (**Figure 5E**). In contrast, MG132 markedly suppressed GFP expression in NF-κB-B7 TRuC-T cells, while NH_4_Cl showed no significant inhibition (**Figure 5G**).

## 4 Discussion

Engineered T cells have demonstrated significant clinical potential in the field of cancer therapy (25, 26). B7-H3 CAR-T cells have shown preliminary efficacy in clinical trials for glioma (24, 27, 28), while the B7-H3-targeted TRuC-T cells generated in this study effectively inhibit glioblastoma cell growth in both *in vitro* and *in vivo* experiments. The expression level of the target antigen on the cell surface is a critical determinant of the recognition and killing efficiency of engineered T cells (29, 30). Compared to CAR-T cells, TRuC-T cells are capable of recognizing and exerting cytotoxic effects against target cells with lower antigen density (31). Our experimental results demonstrate that B7-H3 TRuC-T cells exhibit significantly higher killing efficiency against U-87 cells than against U-251 cells.

Upon target antigen engagement, both NF-κB-B7 TRuC-T and NFAT-B7 TRuC-T cells induced GFP expression, indicating that the cytotoxic activity of TRuC-T cells against target cells is governed by the NF-κB and NFAT signaling pathways. The NF-κB pathway is involved in regulating anti-apoptotic and proliferative functions in CAR-T cells, while the calcium-NFAT signaling axis controls T cell activation—together, these pathways coordinately modulate the functional status of engineered T cells (32–34). Using the NF-κB-B7 TRuC and NFAT-B7 TRuC reporters, we observed that TRuC molecules were downregulated from the T cell surface upon antigen stimulation, with a portion undergoing internalization—a process that was partially reversed by NH_4_Cl and MG132. Consistent with these findings, CAR molecules are known to internalize and degrade upon co-culture with target cells, resulting in reduced surface CAR expression (35); this downregulation often renders this subset of cells difficult to accurately analyze by flow cytometry, leading to their underappreciation in functional assessments. The enhanced killing efficiency of B7-H3 TRuC-T cells against U-87 cells can be attributed to the synergistic effects of reduced TRuC loss from the cell surface and diminished NFAT signaling activity, which in turn alleviated TRuC-T cell exhaustion.

The B7-H3 TRuC construct incorporating NF-κB/NFAT response elements generated in this study serves as a tool to evaluate target antigen expression on the cell surface and to predict whether B7-H3 TRuC-T cells can recognize and eliminate the corresponding target cells. Compared to established visualization techniques such as microfluidic droplet systems, three-dimensional real-time imaging, or single-cell transcriptome sequencing (36), this NF-κB/NFAT-GFP reporter system provides a simple and semi-quantitative platform for *in vitro* assessment of TRuC-T cell functionality. By reporting activation status through endogenous signaling pathways upon antigen recognition, this system enables real-time dynamic monitoring and supports high-throughput screening under standard laboratory conditions, significantly reducing both equipment requirements and operational costs. Nevertheless, this platform has certain limitations: the time delay of several hours between NF-κB/NFAT activation and GFP expression may preclude the detection of early transient signaling events such as immune synapse formation or calcium flux. Additionally, endogenous TCR signaling or other receptors may potentially activate the reporter system, necessitating rigorous control experiments for validation (37–39).

The NFAT family members exert distinct and non-redundant functions in T cell regulation: NFATc1 is predominantly associated with cytotoxic effector programs, while NFATc2 has been linked to the induction of T cell exhaustion (40). Accordingly, NFAT transcriptional element activation cannot be simply categorized as either intrinsically favorable or unfavorable; rather, its functional consequences are critically shaped by the strength and persistence of the upstream signal. Within the context of our short-term co-culture experiments, the enhanced GFP expression observed in response to KNS89 cells most likely signifies rapid TRuC-T cell activation. Nevertheless, it is important to recognize that, in the setting of sustained low-density antigen exposure typical of the tumor microenvironment, NFAT signaling might produce fundamentally divergent outcomes. Furthermore, the density and topological distribution of target antigens on the cell membrane have been established as key determinants of CAR-T cell functional responsiveness—exemplified by CD19 internalization following CAR engagement, which dampens downstream signaling (41). In line with this complexity, our data revealed that KNS89 and U-251 cells, despite expressing lower levels of B7-H3, triggered stronger NFAT-B7 reporter activation. Although the precise mechanisms accounting for this counterintuitive observation remain to be fully elucidated, we posit that inter-cell-line variations in antigen membrane organization and/or immune synapse formation efficiency may contribute to this phenomenon—a hypothesis that awaits direct experimental validation.

This study has several limitations. First, the *in vivo* experiments used immunodeficient xenograft models, which do not fully capture the immune microenvironment, anatomical constraints, or antigen heterogeneity of human glioblastoma. Second, most *in vivo* studies relied on subcutaneous or disseminated tumor models rather than orthotopic intracranial glioblastoma models. Third, the precise upstream molecular events that trigger NF-κB or NFAT activation and thereby influence the cytotoxic activity of B7-H3 TRuC-T cells have yet to be fully characterized, and future studies are required to address this knowledge gap. Fourth, the quantitative threshold of surface B7-H3 molecules needed to engage the NF-κB/NFAT transcriptional elements and drive GFP expression remains to be determined.

In summary, our results establish that B7-H3 TRuC-T cell function is jointly governed by NF-κB and NFAT signaling. Importantly, the NFAT-B7-T reporter platform represents a preliminary strategy to assess B7-H3 tumor antigen abundance, suggesting its potential utility as a reference for the preclinical screening and prediction of therapeutic efficacy in GBM.

## Data Availability

The original contributions presented in the study are included in the article/**Supplementary Material**. Further inquiries can be directed to the corresponding authors.
