# Supplementary material for: NF-κB/NFAT signaling contributes to B7-H3 TRuC-T cell cytotoxicity and supports a reporter platform for glioblastoma immunotherapy
Source: Front Immunol. 2026 Jul 30;17:1876463. doi: 10.3389/fimmu.2026.1876463 (PMC13469471; doi:10.3389/fimmu.2026.1876463)
Supplement: Supplementary file 1 [file DataSheet2.pdf]

通知书

申请人 高基民

你提交的项目： 利用CD3z增强靶向B7-H3 TRuC-T细胞对实体瘤治疗效果的初步研究 （申请编号： 18107 ）中关于动物实验的方案，经我委员会审核，符合要求，予以初审通过。 伦理批号为: wydw2025-0368 .

特此通知。

（注：此通知书仅限于实验方案使用，不得用作其他地方。）

温州医科大学实验动物伦理委员会

温州医科大学实验动物中心（代章）

2025 年 06 月 26 日
