# Supplementary material for: NF-κB/NFAT signaling contributes to B7-H3 TRuC-T cell cytotoxicity and supports a reporter platform for glioblastoma immunotherapy
Source: Front Immunol. 2026 Jul 30;17:1876463. doi: 10.3389/fimmu.2026.1876463 (PMC13469471; doi:10.3389/fimmu.2026.1876463)
Supplement: Supplementary file 2 [file DataSheet3.pdf]

Notification

<u>Applicant: Gao Jimin</u>

The animal experiment protocol submitted for your project: Preliminary Study on Enhancing the Therapeutic Efficacy of Targeted B7-H3 TRuC-T Cells Against Solid Tumors Using CD3z (Application Number: 18107) has been reviewed by our committee.

<u>The application meets the requirements and has passed the preliminary review. The ethical approval number is: wydw2025-0368.</u>

It is hereby announced 。

(Note: This notice is intended solely for use in the experimental protocol and may not be used elsewhere.)

Wenzhou Medical University Experimental Animal Ethics Committee

Wenzhou Medical University Experimental Animal Center

26 June 2025
