# Supplementary material for: NF-κB/NFAT signaling contributes to B7-H3 TRuC-T cell cytotoxicity and supports a reporter platform for glioblastoma immunotherapy
Source: Front Immunol. 2026 Jul 30;17:1876463. doi: 10.3389/fimmu.2026.1876463 (PMC13469471; doi:10.3389/fimmu.2026.1876463)
Supplement: Supplementary file 3 [file Table2.docx]

**NF-κB-responsive transcriptional elements：**5′-GGGAATTTCCGGGGACTTTCCGGGAATTTCCGGGGACTTTCCGGGAATTTCC-3′

**NFAT-responsive transcriptional elements：**

5′-GGAGGAAAAACTGTTTCATACAGAAGGCGTGGAGGAAAAACTGTTTCATACAGAAGGCGTGGAGGAAAAACTGTTTCATACAGAAGGCGT-3′

**mini-promotor：**

5′-TAGAGGGTATATAATGGAAGCTCGAATTCCAG-3′

**B7-H3 TRuC：**

5′-ATGGCTCTGCCCGTGACCGCTCTGCTCCTGCCCCTCGCTCTCCTGCTGCACGCCGCTAGACCCGAGATCGTGCTGACCCAAAGCCCTGCCACCCTCTCCCTGAGCCCCGGGGAAAGAGCCACCCTGAGCTGCAGAGCTAGCAGCAGACTGATCTACATGCACTGGTATCAGCAGAAGCCCGGCCAAGCCCCTAGACCACTGATCTACGCCACAAGCAACCTGGCTAGCGGCATCCCCGCTAGATTCAGCGGCAGCGGCAGCGGCACCGACTTCACCCTGACCATCAGCAGCCTGGAGCCCGAGGACTTCGCCGTGTACTACTGTCAGCAGTGGAACAGCAACCCCCCCACCTTCGGCCAAGGCACCAAGGTGGAGATCAAGAGAACCGGGGGGGGCGGCTCCGGGGGCGGGGGCAGCGGCGGGGGCGGCAGCCAAGTGCAGCTGGTGCAGAGCGGCGCCGAGGTGAAGAAGCCCGGCAGCAGCGTGAAGGTGAGCTGCAAGGCTAGCGGCTACACCTTCACCAACTACGTGATGCACTGGGTGAGACAAGCCCCCGGCCAAGGCCTGGAGTGGATGGGCTACATCAACCCCTACAACGACGACGTGAAGTACAACGAGAAGTTCAAGGGCAGAGTGACCATCACCGCCGACGAGAGCACAAGCACCGCCTACATGGAGCTGAGCAGCCTGAGAAGCGAGGACACCGCCGTCTACTACTGTGCTAGATGGGGCTACTACGGCAGCCCCCTGTACTACTTCGACTACTGGGGCCAAGGCACACTGGTGACCGTCAGCAGCGGTGGAGGCGGCAGTGGCGGAGGTGGGAGCGGAGGGGGCGGTTCCGGTGGCGGGGGATCTGATGGTAATGAAGAAATGGGTGGTATTACACAGACACCATATAAAGTCTCCATCTCTGGAACCACAGTAATATTGACATGCCCTCAGTATCCTGGATCTGAAATACTATGGCAACACAATGATAAAAACATAGGCGGTGATGAGGATGATAAAAACATAGGCAGTGATGAGGATCACCTGTCACTGAAGGAATTTTCAGAATTGGAGCAAAGTGGTTATTATGTCTGCTACCCCAGAGGAAGCAAACCAGAAGATGCGAACTTTTATCTCTACCTGAGGGCAAGAGTGTGTGAGAACTGCATGGAGATGGATGTGATGTCGGTGGCCACAATTGTCATAGTGGACATCTGCATCACTGGGGGCTTGCTGCTGCTGGTTTACTACTGGAGCAAGAATAGAAAGGCCAAGGCCAAGCCTGTGACACGAGGAGCGGGTGCTGGCGGCAGGCAAAGGGGACAAAACAAGGAGAGGCCACCACCTGTTCCCAACCCAGACTATGAGCCCATCCGGAAAGGCCAGCGGGACCTGTATTCTGGCCTGAATCAGAGACGCATCTGA-3′

**Supplementary data1 The nucleic acid sequences of NF-κB-responsive transcriptional elements , NFAT-responsive transcriptional elements ,mini-promotor and B7-H3 TRuC.**

**Supplementary Figure 1** **(A)** Evaluation of GSC cell killing by B7-H3 TRuC-T cells using flow cytometry(n = 3,independent experiments). Data are presented as mean ± SD. Two-way ANOVA with Tukey’s multiple comparisons test was used to test for statistical significance in figure. **p* <0.05, ***p* <0.01, ****p* <0.001, *****p* <0.0001.

**Supplementary Figure 2** NF-κB/NFAT-B7-T cells were co-incubated with astrocytes and endothelial cells for 6 h, and the GFP expression efficiency in TRuC-T cells was evaluated by flow cytometry (n = 3,independent experiments). Data are presented as mean ± SD. Two-way ANOVA with Tukey’s multiple comparisons test was used to test for statistical significance in figure.

**Supplementary Figure 3** (**A)** B7-H3 TRuC-T cells, NF-κB-B7-T cells, and NFAT-T cells were generated, and their manufacturing efficiency was assessed by flow cytometry on day 7 post‑transduction (n = 4, each dot represents an individual donor). (**B)** Mock-T cells were used to normalize the expression levels of B7-H3 TRuC-T cells, NF-κB-B7-T cells, and NFAT-T cells to a comparable range. Total RNA was then extracted, and the mRNA levels of WPRE derived from the lentiviral vector were quantified by qPCR (n = 3, each dot represents an individual donor). (**C)** Mock-T cells, B7-H3 TRuC-T cells, NF-κB-B7-T cells, and NFAT-T cells were co‑incubated with U‑87 cells for 12 h. A luciferase‑based cytotoxicity assay was performed to compare the functional differences among B7-H3 TRuC-T cells harboring NFAT or NF-κB transcriptional response elements (n = 3, independent experiments). (**D)** After normalization of expression levels using mock-T cells, 0.5 × 10⁶ TRuC-T cells were co‑incubated with 0.1 × 10⁶ U‑87 cells. Cell counts were manually determined on days 2, 4, and 6, and the fold change relative to day 0 (set as 1) was calculated to monitor cell proliferation over time (n = 3, independent experiments). (**E)** Schematic representation of the sequential stimulation assay. TRuC-T cells were co-incubated with U-87 target cells at an effector-to-target ratio of 1:1. Proliferation, target cell lysis, and surface PD-1 expression were monitored at indicated time points (n=3, independent experiments).. **(F)** Cytolytic activity of B7-TRuC-T, NF-κB-B7-TRuC-T, and NFAT-B7-TRuC-T cells against U-87 target cells. **(G)** Proliferative capacity of the indicated TRuC-T cell variants, measured by cell counting. **(H)** Surface PD-1 expression levels on B7-TRuC-T, NF-κB-B7-TRuC-T, and NFAT-B7-TRuC-T cells. **(I)** GFP expression profiles in NF-κB-B7-TRuC-T and NFAT-B7-TRuC-T cells upon repeated stimulation. Data are presented as mean ± SD. One-way ANOVA with Tukey’s multiple comparisons test was used to test for statistical significance in A and B, two-way ANOVA with Tukey’s multiple comparisons test was used to test for statistical significance in C , D , F , G , H and I.**p* <0.05, ***p* <0.01, ****p* <0.001, *****p* <0.0001.

**Supplementary Figure 4** Generation of B7-H3 TRuC-GFP T cells. (A) Schematic diagram of the B7-H3 TRuC-GFP construct. The B7-H3 TRuC was linked to GFP via a SGGGGS peptide linker. (B) Evaluation of B7-H3 TRuC-GFP T cell generation efficiency by flow cytometry (n = 3, independent experiments).

Supplementary Figure5 Gating strategy for the flow cytometry data shown in Figure 1. (A), Corresponds to Figure 1B–C, based on donor 1 on day 5. (B), Corresponds to Figure 1D, based on donor 3 (B7-H3 TRuC group). (C), Corresponds to Figure 1E, based on donor 1 (B7-H3 TRuC group).

Supplementary Figure6 Gating strategy for the flow cytometry data shown in Figure 2. (A), Corresponds to Figure 2E, based on the unstimulated B7-H3 TRuC-T group (1st).

Supplementary Figure7 Gating strategy for the flow cytometry data shown in Figure 3. (A), Corresponds to Figure 3B, based on the NF-κB-G-M(F) group. (B), Corresponds to Figure 3D, based on the NF-κB-B7 TRuC-T group on day 7. (C), Corresponds to Figure 3E, based on the PMA-stimulated NF-κB-B7 TRuC-T cell group. (D), Corresponds to Figure 3F, based on the NFAT-B7 TRuC-T cell group stimulated with 500 ng/mL B7-H3 protein. (E),Corresponds to Figure 3G, based on the unstimulated NFAT-B7 TRuC-T cell group at 6 h.

.

Supplementary Figure8 Gating strategy for the flow cytometry data shown in Figure 4. (A), Corresponds to Figure 4A, based on the first group of NFAT-B7-T cells (blank control). (B), Corresponds to Figure 4B, based on the first group of NFAT-B7-T cells (blank control). (C), Corresponds to Figures 4D and 4E, based on NFAT-B7-T cells co-incubated with U-87 cells. (D), Corresponds to Figure 4F, based on NFAT-B7-T cells co-incubated with U-87 cells. (E), Corresponds to Figure 4G, based on NFAT-B7-T cells co-incubated with U-87 cells. (F），Corresponds to Figure 4I, based on NFAT-B7-T cells co-incubated with U-87 cells.(G,H) Corresponds to Figure 4J, based on NFAT-B7-T cells co-incubated with U-87 cells.

Supplementary Figure 9 Gating strategy for the flow cytometry data shown in Figure 5. (A), Corresponds to Figure 5A, based on NF-κB-B7-T cells co-incubated with U-87 cells. The gating strategy for Figure 5C is the same as that for Figure 5A.
